# U.S. food policy to address diet-related chronic disease

**DOI:** 10.3389/fpubh.2024.1339859

**Published:** 2024-05-16

**Authors:** Emily D. Matthews, Emma L. Kurnat-Thoma

**Affiliations:** ^1^Emergency Department, Holy Cross Hospital, Holy Cross Health, Silver Spring, MD, United States; ^2^Georgetown Institute for Women, Peace and Security, Walsh School of Foreign Service, Georgetown University, Washington, DC, United States; ^3^Precision Policy Solutions, LLC, Bethesda, MD, United States

**Keywords:** food policy, food insecurity, SNAP benefits, sugar sweetened beverages, sustainable food systems, climate smart agriculture, food is medicine

## Abstract

Poor diet is the leading cause of mortality in the U.S. due to the direct relationship with diet-related chronic diseases, disproportionally affects underserved communities, and exacerbates health disparities. Evidence-based policy solutions are greatly needed to foster an equitable and climate-smart food system that improves health, nutrition and reduces chronic disease healthcare costs. To directly address epidemic levels of U.S. diet-related chronic diseases and nutritional health disparities, we conducted a policy analysis, prioritized policy options and implementation strategies, and issued final recommendations for bipartisan consideration in the 2023–24 Farm Bill Reauthorization. Actional recommendations include: sugar-sweetened beverage taxation, Supplemental Nutrition Assistance Program (SNAP) fruit and vegetable subsidy expansion, replacement of ultra-processed foods (UPF) with sustainable, diverse, climate-smart agriculture and food purchasing options, and implementing “food is medicine.”

## 1 Introduction

Approximately one million people die annually from diet-related chronic diseases in the United States (U.S.), and these numbers are rising due to prolonged COVID-19 impacts (1). Poor diet is the leading cause of U.S. mortality which is directly related to malnutrition and chronic diseases including type 2 diabetes (T2D), cardiovascular disease (CVD), obesity, and some cancers (2, 3). Almost half of U.S. adults have pre-diabetes (38%) or diabetes (11.3%), with most cases as T2D (4). In 2022, 126.9 million Americans 20 years and older have some form of CVD, comprising approximately 37% of the U.S. population (5). CVD is the leading cause of death for men and women in the U.S., accounting for 695,000 total deaths in 2021 (6). In 2021, diabetes was the eighth leading cause of mortality in the U.S., resulting in 103,294 deaths (7). Diet-related chronic diseases and malnutrition disproportionally affect underserved communities in areas of higher poverty, who do not have access to affordable, healthy, and nutritious foods (8). Access to healthy nutritious foods is an essential social determinant of health (SDOH), and is heavily influenced by local environments and community infrastructure.

The U.S. needs innovative approaches and evidence-based policies to reduce consumption of unhealthy foods and to support accessibility of healthy climate-smart foods and nutritional therapies as part of the “food as medicine” movement. Implementing policies improving American nutrition will significantly impact public health by reducing rates of diet-related chronic diseases, nutrition inequity, and its related healthcare spending. These foods must be widely available, environmentally sustainable, and culturally relevant for vulnerable and impoverished communities.

The current bipartisan 2023–24 U.S. Farm Bill reauthorization presents an opportune time to enact effective and impactful policies to improve America’s nutrition and health. Initially designed in 1933 to address agriculture, the Farm Bill is an omnibus bill which evolved over time to support affordability of healthy foods (9). Significant milestones include the Food Stamp Act of 1964 to provide financial assistance for food security which evolved into the Supplemental Nutrition Assistance Program (SNAP). SNAP is the largest U.S. food assistance program and provides monthly benefits for approximately 40 million low-income Americans, primarily for children, older adults and individuals with disabilities (10). Congress reauthorizes SNAP every 5 years as part of the Farm Bill, which provides policymakers a valuable opportunity to improve healthy nutrition and reduce U.S. diet-related chronic diseases by helping U.S. families gain more access to nutritious foods (11, 12). Effective SNAP implementation must emphasize nutrition equity to reduce diet-related health disparities of low-income Americans (13). For example, lower-income communities consume greater amounts of ultra-processed foods (UPF) resulting in lower quality diets due to their longer shelf life, wide accessibility and lower costs, making them a convenient option for food insecure households and a continuous driver of diet-related chronic disease (14–16). Low income communities are highly targeted by UPF supply chains and are subject to aggressive marketing (16). Consequently, UPF’s are a dominant method of combatting food insecurity, which generates a vicious addiction cycle of maladaptive behavioral preferences as a function of their environmental context (14, 15). Thus, SNAP participants struggle more than higher income groups to meet the U.S. Department of Agriculture (USDA) dietary guidelines and achieve a nutritious diet. Table 1 presents a summary of several SNAP-related programs, the most directly relevant being GusNIP, to support incentivization of fruit and vegetable (F/V) production, distribution, purchasing, and consumption in low-income communities (17).

**Table 1:** Selected key Farm Bill provisions for supporting fruits and vegetables (F/V, specialty crops).

Program	Farm bill year	Grant amount	Description
FCIP Federal Crop Insurance Program	2018	$22 billion in FY21 insurance liability protection for specialty crops	Reimbursement for high-value specialty crops from physical losses for insurable indications, including: adverse weather conditions (i.e., hail, frost, freeze, wind, drought and floods), earthquake, unexpected pest or disease without sufficient prevention methods, some irrigation failures, wildfires and volcanic eruptions, etc.
FFVP Fresh Fruit and Vegetable Program	2018	$233.1 million in FY22	Started in 2002 to increase F/V consumption in low-income elementary schools and students, associated with food waste plate reductions, obesity reduction.
FINI Food Insecurity Nutrition Incentive Program	2014	$85.6 million in FY15-18	Initial pilot program created to incentivize F/V purchasing in low-income communities among SNAP participants, measured by benefits usage and F/V consumption rates.
FPDP Food Purchase and Distribution Program	2018	$2.3 billion in FY19-FY20	Trade mitigation funds to assist farmers suffering damage due to unjustified trade retaliation from foreign entities for domestically grown F/V and to support emergency food assistance via food banks, pantries, networks (TEFAP).
GusNIP Gus Schumacher Nutrition Incentive Program	2018	$270 million since FY19	Expanded FINI from 2014. Low-income F/V grants and Medicaid nutrition prescriptions; mandatory growth $45–56 million over 5 years FY19-FY23 in all 50 states and participating territories.
SCRI Specialty Crop Research Initiative	2018	$80 million in FY23	Competitive grants supporting improved agricultural management of specialty crops: fruits, vegetables, dried fruit, tree nuts and others. Focus areas include: improved crop resilience through genetics, genomics, non-toxic control of pests and diseases, increased production yields and higher quality at decreased costs, food safety and new tech innovations.
SNAP Supplemental Nutrition Assistance Program	2018	$159 billion in FY22 (all nutrition funds)	Comprises the majority of federal nutrition assistance expenditures, formerly known as ‘Food Stamps’. Provides EBT benefits to low-income households, individuals and families to purchase food at SNAP-authorized retailers. Additional incentives for F/V such as ‘double bucks’ and online purchases at selected retail hubs.

Not a comprehensive Farm Bill F/V specialty crop allocation list. All funding statistics are selected from online USDA funding program allocation hubs per FY indicated. Reviewed in Congressional Budget Office baseline projections where available.

In September 2022, the Biden-Harris administration released the *National Strategy on Hunger, Nutrition, and Health*, representing the first domestic U.S. policy conference on this topic in over 50 years (18). The federal government’s bipartisan multisector plan to reduce diet-related diseases, improve nutrition, fight hunger and food insecurity, and reduce health disparities comprises five-pillars. Table 2 summarizes each of the five pillars published in the White House’s strategy and highlights selected components relevant to decreasing U.S. diet-related chronic disease (19). The 2023–24 Farm Bill reauthorization provides a valuable post-COVID-19 policy opportunity to improve U.S. health via the participation of a wide variety of strategies, sectors and to strengthen SNAP benefits modalities, coverage and efficacy for low-income Americans (20–22).

**Table 2:** Selected components for the 2022 U.S. national strategy to end hunger and build healthy communities.

Pillar	Strategy overview for reducing hunger, increasing healthy eating and physical activity by 2030 for decreased diet-related diseases and disparities
1. Improving food access and affordability	Advancing economic stability and security to increase access to free nourishing school meals, providing electronic food benefits to children, expanding SNAP eligibility for underserved populations. Making it easier for everyone in low-income urban, suburban, rural and Tribal communities and Territories to access nutritious food in their geographical cultural and social determinants (SDOH) contexts.
2. Integrating nutrition and health	Ensuring nutrition and food security is prominently featured in disease prevention, management across the human lifespan. Piloting coverage of medically tailored meals in Medicare/Medicaid via “food as medicine” for diet-related diseases, testing Medicaid coverage of multi-modal nutrition education, health promotion supports and demonstration projects, including nutrition and obesity counseling.
3. Empowering all consumers to make and have access to healthy choices	Foster environments that enable all people to easily make informed, healthy choices, increase access to healthy foods at home, in the workplace, and at schools. Investing in public awareness, education campaigns that are appropriate and culturally tailored for underserved communities. Front of package food labels and e-labels, precision nutrition criteria for ‘healthy’ food claim use, expanding SNAP incentives for fruits, vegetables, reduction of added sodium and sugar via voluntary industry commitments, ensuring safe, resilient food supply and adequate resources for infectious disease pathogen detection and surveillance.
4. Supporting physical activity for all	Making it easier for Americans to be more physically active by ensuring safe physical activity spaces by connecting to more parks and trails, increasing awareness of the benefits of exercise, and conducting additional research on its accurate measurement and health effects across a variety of health promotion and disease management contexts, particularly digital tools. Scaling up of DHHS Centers for Disease Control and Prevention (CDC) to all states and territories, connecting Americans to parks and outdoor spaces, fully funding and promoting the *Physical Activity Guidelines for Americans*.
5. Enhancing nutrition and food security research	Strengthening nutrition and food security research to improve nutrition metrics and measurement methods including digital and e-tools, provide foundational knowledge for evidence-based policymaking to advance nutritional equity, improve healthy food access, resilient food systems, and reduce disparities for underserved populations. Nutritional science, climate-smart, climate resilient agriculture, sustainable food systems, health promotion research and education, workforce development, diversity for innovative advancements spanning bench to bedside including: precision medicine, precision health, artificial intelligence, machine learning, genomic and -omic sequencing, gene and environmental interactions for a variety of SDOHs, agricultural, climate, vendor/retailer and technical contexts.

The goal of this project was to conduct a policy analysis, identify four key strategic food policy insight areas to support equitable U.S. food policy supporting the National Strategy, and formulate options and recommendations for further consideration in this year’s 2023–24 Farm Bill Reauthorization. Our policy analysis question was: what policy options can strengthen U.S. nutrition to promote a healthy, equitable, climate-smart, sustainable food system and reduce diet-related chronic disease?

## 2 Methods

**Figure 1:**
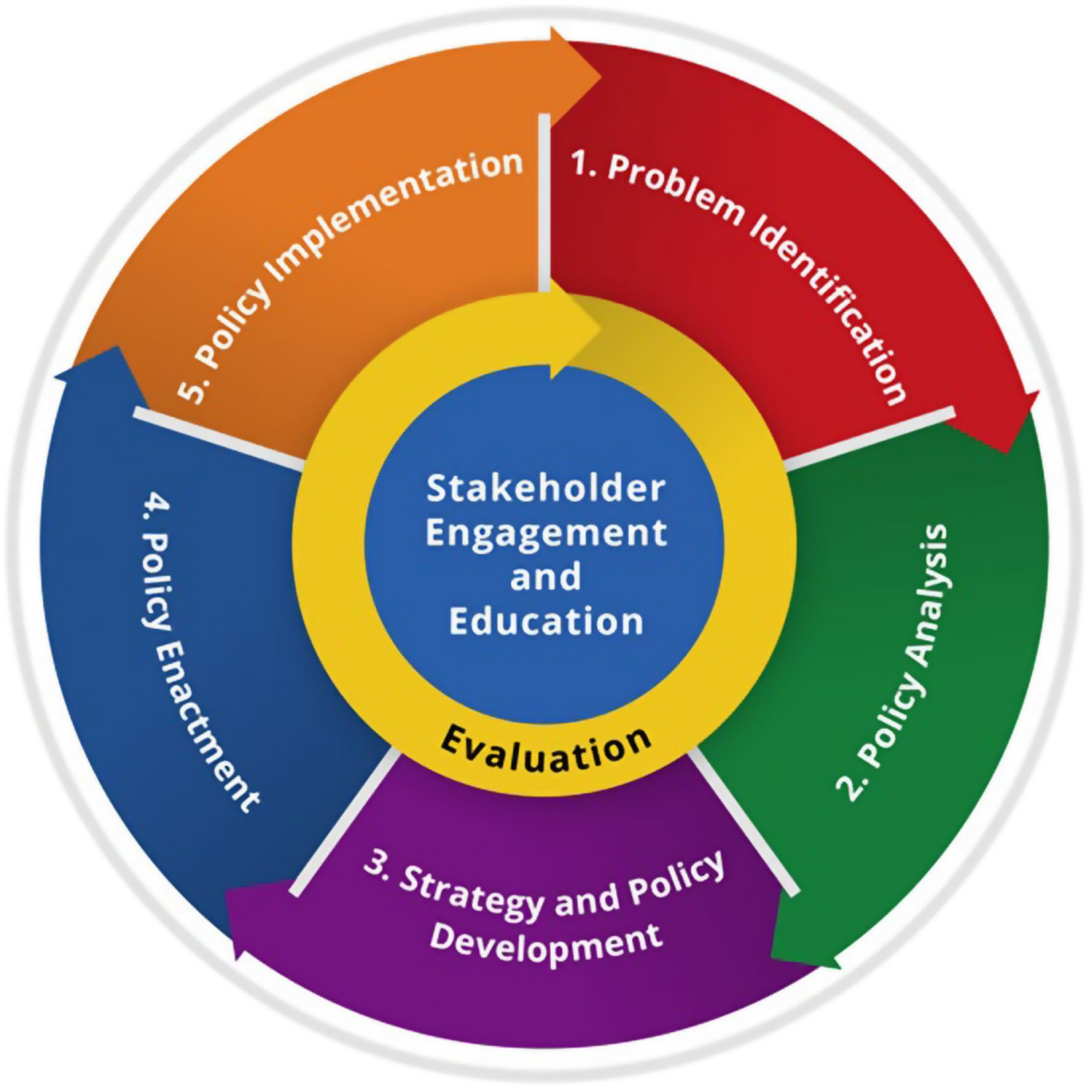
The CDC policy analytical framework. This project used domains I, II, III of Centers for Disease Control and Prevention (CDC) policy analytical framework process. The CDC policy analysis framework is available in the public domain from the U.S. DHHS (23).

We performed a policy analysis in Spring 2022 using Centers for Disease Control and Prevention’s (CDC) Policy Analysis Framework tool, depicted in Figure 1 (23). The CDC plays a significant and non-biased role in analyzing policy options for public health problems through the lens of health, economic and budgetary impacts. The Policy Analysis Framework Tool, developed in 2013 by CDC’s Office of Policy, Performance, and Evaluation, aims to strengthen local community through national level policy analysis and strategy planning processes. It does this by appropriately identifying and prioritizing the most impactful public health problems; analyzing and researching policy, health impacts, economic and budgetary gaps to identify possible solutions; developing evidence-based policy intervention options; identifying strategic partnerships that can effect change; aligning key stakeholders for political feasibility; and translating science into practical use implementation and enactment strategies to achieve domestic U.S. health goals.

We completed the first three structured domains of the CDC Policy Analysis Framework: problem identification; policy analysis and assess, prioritize available options; and, final policy recommendation for implementation. For problem identification, we conducted a literature review of U.S. food policy topics for focused development, their primary public health impacts, and contributing factors using PubMed, Ovid, CINAHL, Google Scholar, key federal agencies, agency databases and gray literature reports (i.e., Congressional Research Service, U.S. Government Accountability Office, Congressional Budget Office, Centers for Disease Control and Prevention, National Institutes of Health, U.S. Department of Agriculture, etc.). Once the problem and contributory components were identified, additional agency information on key programs, costs, outcome metrics and population statistics were obtained to generate an accurate problem background context and U.S. food policy landscape. Several periods of re-review were conducted (Fall 2022, Spring 2023, Summer 2023) to update evidence, refine selected food policy insight areas, and clarify domestic political landscape issues for which to address U.S. diet-related chronic diseases and nutritional equity. We developed, rated and prioritized policy options based on health, economic and budgetary impacts, political feasibility and analyzed implementation strategy pros/cons across four insight areas: (1) sugar sweetened beverages; (2) food insecurity; (3) reducing UPFs by incentivizing healthy F/V from environmentally sustainable climate-smart agriculture sources; and (4) prioritizing and expanding “food as medicine” nutrition prescriptions. Final policy recommendations and implementation strategies were described in accordance with the evidence.

## 3 Policy landscape insights and implications

### 3.1 Policy insight 1 – Sugar sweetened beverages (SSBs)

Sugar-sweetened beverages (SSBs) are considered any liquid with added forms of sugar including regular soda, fruit drinks, sports drinks, energy drinks, sweetened waters, and sweetened coffee and tea drinks (24). According to the 2020–2025 Dietary Guidelines for Americans, added sugars should be limited to less than 10% of daily caloric intake (25). However, SSBs remain inexpensive, widely consumed, are the leading source of modifiable sugar consumption in the U.S. and are linked to increased body mass index and obesity, preventable death/disability, and global diet-related chronic diseases such as T2D and CVD (26–28). U.S. SSB consumption is higher in males, young adults ages 20–39 years, non-Hispanic Black individuals and Mexican Americans, and adults with low incomes below 130% of the poverty line (24, 28). DiFrancesco et al.’s comprehensive trend analysis of sugar intake trends in 72,829 U.S. National Health and Nutrition Examination Survey (NHANES) adult participants from 2001 to 2018, indicated although SSB consumption trends decreased over time, they remained the most significant source of added sugar intake at 37% in 2017–2018 (28). Consumption of artificially sweetened beverages (ASBs) are also associated with developing T2D, CVD, obesity, hypertension and all-cause mortality; however, more scientific evidence is needed to understand conflicting associations, effects strengths and possible carcinogenic impacts at high doses (29).

### 3.2 Policy insight 2 – Food insecurity

Food secure households maintain consistent food access for a healthy and active lifestyle. In 2022, 12.8% of U.S. households were food insecure, and even reached as high as 20.4% during the lockdown portions of COVID-19 (April 2020), demonstrating increased vulnerability during times of economic disruption and uncertainty (30). Of the 17 million Americans that were food insecure in 2022, they met the thresholds of low (10.2 million) and very low food security (6.8 million) (30). Low food security leads to disruptions in eating patterns and a need for federal assistance such as SNAP benefits, while very low food security results in disrupted patterns and reduced food intake (30). In 2022, 11.7 million adults and 783,000 children, faced very low food security, where food intake steadily decreased throughout the year due to direct lack of money and resources for which to purchase healthy food (30). According to a 2019 national population-based CDC survey, approximately 90% of all Americans *do not* consume the recommended amounts of F/V (31, 32). Common U.S. barriers to purchasing, preparing and consuming F/V are more pervasive in low resource communities with lower food security and are high priority areas for benefit design prioritization (33). Food insecurity is linked to additional healthcare from skipping and delaying medications and healthcare visits, further perpetuating the problem of poor health (34). U.S. food insecurity demonstrates well-characterized rural and urban geographic, regional patterns with disproportionate impacts on high poverty counties with African American, Alaska Native/American Indian, racial and ethnic minority groups (35, 36). During the COVID-19 pandemic, all U.S. counties were impacted by hunger and food insecurity, and after the pandemic, food insecurity rates among African Americans/Black individuals or Latinos were higher than white individuals in nearly all counties (99%) (36). Nine of ten high food insecurity counties are rural; 82% of the highest food insecurity counties are located in the South and directly linked to unemployment and poverty (35, 36). Current 2024–2034 Congressional Budget Office post-pandemic SNAP projections expect participation of 41.1 million individuals and a 17% cost decrease ($23 billion) due to the end of COVID-19 emergency funds supporting additional food benefits to families with children (37).

### 3.3 Policy insight 3 – Ultra-processed foods (UPF) and unsustainable food systems

UPFs are foods that have undergone multiple industrial production processes, include frequent use of cosmetic additives for longer shelf-life and hyper-palatability, and involve synthetic formulations of energy dense ingredients including sugar, salt, and trans fats (38). Population based cross-sectional studies indicate UPFs are common staples of the “Westernized diet” for the past two decades (14, 39, 40). They are often found in “food deserts,” where healthful nutrition is minimal, and “food swamps,” where UPF-market retailers dominate the healthier options (41). UPFs induce a high glycemic response, possess low satiety, and account for more than half the total dietary energy consumed in the U.S. (38). Srour et al. (42, 43) found higher UPF grams per day intake was associated with higher risks of CVD, T2D, coronary heart and cerebrovascular disease. “Westernized” UPF eating patterns have grown in prevalence globally and possess both negative human health and environmental impacts (44). Existing obesity, chronic disease and undernutrition health challenges for which UPF contribute, are significantly compounded by climate change, sustainable agriculture and food production availability. Three factors—undernutrition, obesity and climate are a “synergy of pandemics” (45). To reduce UPFs, harmful production and consumption supply chains must be neutralized, while increasing availability of minimally processed nutritious foods sourced from environmentally sustainable agriculture and climate-smart farming practices. Diets lower in animal products generally emit fewer greenhouse gases (GHG) than meat heavy Western diets. Climate smart and resilient foods feature diet diversification with whole grains, legumes, fruits, and vegetables sourced from environmentally sustainable food systems, reduced meat sources, focus on key energy and nutrient crops (i.e., vitamin B12, folate) and parallel agriculture and livestock designs with minimal food waste losses (46, 47). Nutritious dairy, lean meats, and fresh produce grown in protected conditions and/or distributed through air transportation may have higher total GHG environmental impacts despite being very healthy. Ensuring U.S. food and nutrition policy can synergistically advance improvements in both human and environmental health, simultaneously protects both key determinants of human welfare. Per Willet et al. “food is the single strongest lever to optimize human health and environmental sustainability on Earth” (47).

### 3.4 Policy insight 4 – “Food is medicine” for T2D and CVD

As shown in Table 3, prevalence and financial burdens of T2D, CVD and other diet-related chronic disease has reached epidemic levels; these issues must use a multifactorial approach with diet at the center. “Food is medicine” (or produce prescriptions), is an evidence-based policy approach gaining much interest and favorable feedback from multi-sectoral stakeholders across the political spectrum to facilitate consistent use of USDA-DHHS dietary nutrition guidelines in routine healthcare, especially for those with limited access to healthy foods and health insurance coverage (25, 48). For example, Scrafford et al.’s economic modeling projections estimated $16.7–$31.5 billion healthcare cost savings from conformance with the 2015–2020 U.S. Dietary Guidelines (49). Although protocols vary, several studies indicate U.S. guidelines with greater intakes of whole grains, vegetables, fruits, legumes, nuts and lower intakes of processed foods, meat and poultry, and SSBs, can be tailored to reflect a wide variety of cultural preferences and ethnic traditions (48–50). Community based organizations (CBOs) help ensure appropriateness of “food is medicine” offerings by preserving cultural heritage, while maintaining healthful dietary guidelines and ensuring reduced sugars, saturated fats, and sodium (25, 48–50).

**Table 3:** U.S. financial burden of diet-related chronic disease.

	Type 2 diabetes	Cardiovascular disease
Spending per healthcare dollar	$1 in $4 (most expensive US condition) (74, 75)	$1 in $6–$7 (5, 6, 77, 78)
Population impacted	1 in 10 adults diagnosed (11.3%; CI 10.3–12.5) (4) 37–38.4 million (4, 75)	1 in 20 adults (5%) (5, 6) 127 million (1 or more CVD condition of Heart and/or Blood Vessels) (77)
Direct medical costs	$237 billion (4, 75)	$239.9 billion (5, 6)
Total medical costs (direct, indirect)	$327 billion (75)	$378 billion (5, 6)
Medicare % funding of direct medical costs	61% (4, 75)	54% (79)
Average annual costs per Medicare beneficiary	$5,876 (4, 75) $3,609–$5,283 (out of pocket, OOP) (76)	$18,270 (80) $2,329 (OOP) (80)
Disproportionality affected populations	American Indian/Alaska Native, Hispanic, non-Hispanic Black, and Asian individuals (75)	Non-Hispanic Black individuals; Pacific Islander and Asian American females; American Indian/Alaska Native and Hispanic females (5, 6)

Unless otherwise indicated, statistics are selected from CDC’s population health resources, which contains secondary, tertiary links to the specific research study and/or population database involved (i.e., CDC WONDER, CMS beneficiary, AHRQ MEPS, NHANES, etc.).

“Food is medicine” interventions typically feature prescriptions based on a person’s unique health, lifestyle and diet-related disease medical diagnosis requirements that can be linked to insurance reimbursement, care coordination and case management structures. Specific formats include medically tailored meals (MTM), customized groceries, food insecurity produce prescriptions, precision medicine, precision health, and precision nutrition science. Although these disciplines and interventions can help combat diet-related chronic disease and significantly reduce healthcare costs, sufficient scientific evidence is needed for upscaling reimbursable interventions in clinical and community settings, especially for ethnic minority, vulnerable and underserved populations (48, 51–53). For example, Berkowitz et al. found 50% fewer inpatient admissions and 70% fewer emergency department visits for dual eligible Medicare-Medicaid participants that received MTM for 6 months with an average net savings of $220 per patient (54). An additional study by Berkowitz et al. found MTM service with 10 weekly meals was associated with significantly decreased inpatient admissions, fewer skilled nursing facility admissions, and ~ $753 less medical expenditures per person (55). Nutritionally calibrated “food is medicine” interventions, continue to show significant promise by empowering patients and caregivers to optimally prevent and manage chronic diseases and related complications (48).

## 4 Policy options and final recommendation

Evidence based policy options are summarized next. Table 4 presents the final policy recommendation in addition to detailed implementation advantages, disadvantages.

**Table 4:** Final U.S. food policy recommendation and implementation strategy considerations.

Policy recommendation	Implementation strategy pros	Implementation strategy cons
SSB taxation	Consistent evidence of SSB taxation efficacy in contrast to education and marketing campaigns alone. SSB taxation pilot implementations in various US cities, regions and international cities, countries demonstrate an overall acceptance of this approach (59). Tax revenue has the potential to aid in additional supportive community health promotion initiatives to reduce diet inequity.	Higher taxes may be financially regressive in lower income communities. Vital for tax revenues to be used for targeted health promotion strategies within these communities. Need to accompany SSB taxation with accurate marketing and education for specific demographic groups tailored to risk. Children, adolescents, low-income, and racial minority groups (Black, Hispanic, and AI/AN individuals, etc.) are the most harmed by unhealthy beverages and poor nutrition standards. ASB taxation linked to SSBs show mixed outcomes; ASBs have potential carcinogenic health risks in larger doses.
F/V subsidy	F/V consumption directly addresses both nutritional insecurity and food insecurity among SNAP participants. Expansion of FINI and GusNIP are proven demonstration routes to encourage nutritious food and F/V consumption in low-income communities across the U.S. and its Territories. Every $5 of new SNAP benefit generates $9 economic activity. Program modernization enhancements for access, integrity, technology, and operations efficiency improves SNAP experience for both retailers and participants, particularly for virtual shopping, online grocery stores and e-benefits.	F/V are a specialty crop commodity and require specialized considerations, such as crop insurance, to mitigate climate change disasters. Subsidy does not guarantee behavior change among SNAP participants who choose less healthy options. Eligibility and workforce participation requirements may cause undue participation barriers and should be carefully considered. There can be inflation impacts on SNAP food prices for participants. Health and nutrition literacy—accurate science-based nutrition labeling for healthy foods—for virtual consumers and electronically purchased products may not always be available. Child specific nutrition programs (i.e., WIC, school nutrition programs) are not necessarily aligned with SNAP structures and appropriations, but could still benefit from shared F/V specialty crop commerce supply chains.
Reduce UPF by incentivizing climate-smart foods	Has the potential to promote industry reformulations toward healthy foods, taking advantage of consumer demand and ultimately leading to a lasting, longer-term effect on diet and public health. Climate-smart agricultural production is a tremendous opportunity to support longer-term productivity, prosperity, and resilience of U.S. farms, forest lands and rural communities. Obtaining and setting reasonable GHG, quality standards for sustainability, food-waste reduction in procurements for a wide range of public venues, including healthcare systems. Voluntary multi-sectoral partnerships for climate-smart commodities can be an effective low-cost government strategy. Simultaneously helps reduce GHGs, risk of catastrophic shifts in Earth’s fragile ecosystems while directly impacting diet related chronic disease, a powerful win-win synergy strategy. Directly address food supply inequity by neutralizing food deserts and food swamps.	More difficult to reduce and directly disincentivize ultra-processed foods (UPFs) due to partisan political and corporate climates. UPF, concentrated animal feeding operations, animal source protein, industrial hemp loopholes (non-drug component <0.3% THC) vs. marijuana (>0.3% THC, especially in unregulated states with unclear FDA specs) need to be re-considered in hot spot areas of severe undernutrition, malnutrition, economic poverty, and environmental injustice. Incentives for healthy diet goals must be paired with dramatic reduction in food waste and improved food production practices to achieve safely operating and resilient food systems. Adequate community engagement for crop insurance during shift(s) to climate-smart agriculture production, specialty crops to ensure climate resilience through extreme weather, climate disaster events. Need trade mitigation supports and practical GHG targets. Need cooperative bipartisan networks and alliances to work with industry stakeholders, for addressing the most harmful market impacts on distressed communities and small-medium farmer agriculture sources.
“Food is Medicine”: MTM benefits, research and development; implementation pilots & scale-ups	Provides directly prepared meals to vulnerable patients who are unable to grocery shop and/or cook. Adequate access to nutritious food is a key component of any SDOH framework for promoting human health and preventing, treating human illness. Provides customized diet and health solutions based on a person’s unique biologic makeup, environment, and lifestyle choices. Valuable opportunity for technology innovations to advance digital nutrition and health for underserved and vulnerable populations (i.e., dual eligibles). Diverse cultural traditions and preferences present myriad possibilities for healthy nutrition systems pathways and for cultivating a diverse nutrition science workforce.	Would need direct involvement and facilitation within a healthcare setting, creating another significant step in implementation and potential barriers. Some interventions do not have sufficient scientific evidence and need rapid R/D investment for piloting and strategic interagency coordination for rapid scale up. Must pay particular attention to adequate inclusion of vulnerable and underserved populations in research participation and health insurance benefits reimbursement to fully address the most severe chronic diet-related health inequities. Ethical applications and stewardship of technology advancements in empowering use of digital innovations (i.e., health information, privacy and confidentiality, artificial intelligence, insurance benefits). Need to account for other SDOH factors, including insurance access, housing, poverty, education, and literacy levels, etc.

### 4.1 Policy option 1 – SSB Taxation

Well-designed taxation structures consistently demonstrate evidence of effective SSB consumption decreases contributing to prevention and control of non-communicable diet-related chronic diseases, such that more than 85 countries at national or subnational governance levels use some sort of SSB taxation (56–59). A 20% tax on SSBs, such as that advised by the World Health Organization, can help reduce sugar consumption with little nutritional value by disincentivizing SSB purchases for healthier consumer options, reduce risk of diet-related chronic diseases and strengthen community health (59). For example, Krieger et al. collected data on 7 U.S. cities with a 1–2 cents per ounce SSB excise tax; annual tax revenue was $133.9 million with allocations of $133.2 million back into community infrastructure and programs for a full fiscal year (60). Roberto et al. evaluated a beverage excise tax implemented on both SSBs and ASBs in Philadelphia, resulting in a 51% purchase decrease, but this was offset by increased sales in bordering regions without the tax (61). Price plays a key role in food choice, making SSB tax an important tool to target the largest sugar contributor to the U.S. diet and reduce SSB purchases. Revenue should be allocated into a tax fund with clear specifications, governed for promoting health equity, addressing key SDOH priorities and tailored to community needs.

### 4.2 Policy option 2 – Healthy food subsidies for F/V

Vendors, retailers and small grocers in high poverty environments have lower access to nutrient dense foods from producers (62). A significant obstacle is that current U.S. F/V subsidy programs are piecemeal and not applied to the entire population. Pomeranz et al. examined the feasibility of various national F/V subsidies in existing local programs and their population health outcome impacts and concluded a nationally mandated subsidy can help reduce diet-related chronic disease and U.S. health economic burdens (62). These programs should be broadly expanded in the 2023–24 Farm Bill and integrated into Electronic Benefits Transfer (EBT) modalities while broadly upscaling other innovative digital program administration and online grocery purchase options that work well for consumer preferences, such as the popular F/V ‘double-bucks’ formats (63–65). Ensuring a greater variety of access, ordering and delivery approaches with online modalities, sufficient Broadband infrastructure and retail hub access will be key to advancing innovative SNAP benefit participation and utilization, especially in rural areas featuring complex interactions between individual state, multi-state, federal and commerce networks (62). Supporting and empowering lower income and underserved communities to strengthen access to healthy, fresh and unprocessed foods at affordable prices, with adequate SNAP benefits, in convenient consumer empowerment formats, even during times of market uncertainty and government crisis (i.e., COVID-19), are key to reducing U.S. nutritional and diet-related health disparities (66, 67).

### 4.3 Policy option 3 – Incentivize healthy, environmentally sustainable climate-smart foods

Policy options for government regulations reducing consumption of UPFs and food additives, to strengthen promotion of SNAP benefits for minimally processed F/V from sustainably sourced food systems, would greatly improve nutrition, consumer choices supporting specialty growers, products and crops (68, 69). Adherence to mandatory or recommended industry quality standards for limits on unhealthy ingredients and formulations using trans fats, salt, and sugar provide a well-defined approach and yield predictable results based on political feasibility. Although no singular policy on its own can change the U.S. food system to prevent T2D and CVD, thoughtfully designed interconnected policies that mutually reinforce UPF reduction and consumption in vulnerable populations and environmental contexts, are increasingly recognized as a valuable strategy for promoting healthier diets and nutritionally equitable, food secure landscapes (44). Policies to reduce and mitigate UPF harms can be enacted in a step-wise approach, and include front of packages (FOP) warning labels, marketing limitations and ingredient restrictions, particularly for young children, adolescents (44, 70, 71). Several countries have adopted the use of a warning label on UPFs, directly informing consumers leading to a decrease in consumption (44). Since most U.S. rural areas with greater poverty levels rely on mid-size and family farm economies, government must also foster an equitable, climate-smart food system by integrating both human health and environmental sustainability priorities to be sufficiently resilient to withstand natural disasters, adverse weather events and agricultural conditions (72, 73). Climate-smart and sustainable diets should consist largely plant based foods, unsaturated fats, reduced amounts of animal food sources and limited UPFs, trans fats, refined grains, added sugars (45, 47). Government-promoted shifts and bipartisan multi-sectoral collaborations can be harnessed to help ensure the U.S. food system to achieve sustainability, minimize waste byproducts, and stay within necessary boundaries to protect domestic and global earth systems (47, 72). Critical boundaries for consideration include GHG emissions targets, practical feasibility for cropland usage and climate disaster impacts, freshwater usage, nitrogen and phosphorus cycling, and biodiversity loss mitigation.

The EAT-Lancet Commission Report outlines an integrated global framework with quantitative scientific targets for both healthy diets and sustainable food production to avoid severe environmental degradation and prevent approximately 11 million human deaths annually (47). This landmark report recommends regionally tailored healthy diets within optimal caloric intake guidelines comprised of a diversity of plant-based foods and unsaturated fats, while limiting amounts of animal source foods, refined grains, highly processed foods and added sugars to support large scale production and sustainability models.

### 4.4 Policy option 4 – Incentivize “food is medicine” interventions

Evidence-based “food is medicine” interventions and nutrition access including MTM, medically tailored groceries, precision medicine, nutrition, health and others should be prioritized for research funding and implementation in the U.S. healthcare system (18–20, 52, 53). Risk predictions, pilot interventions and community based supports for Medicare/Medicaid high cost/high need patients (dual-eligibles) and other at-risk populations shouldering high burdens of chronic diet-related illnesses should be especially prioritized if unable to cook or shop for themselves. Patients with high cost chronic conditions T2D, CVD and other diet-related chronic illness should be screened by CBOs within the healthcare SDOH contexts for “food is medicine” intervention eligibility (6, 48, 74). Culturally tailored interventions and community preferences must be honored in clinical and scientific research initiatives. However, Medicare, Medicaid and private insurers should reimburse CBOs, providers, and retailers for “food is medicine” interventions and services to ensure they are available for as many as possible. Healthcare providers should receive reimbursements for screenings according to the strength of evidence from robust federal scientific initiatives, guideline recommendations and population level nutritional surveillance programs.

## 5 Conclusion

U.S. nutrition and diet-related chronic diseases are at epidemic levels, were greatly exacerbated by COVID-19, and continue to extensively evolve to accommodate consumer preferences and food system supply/demand workflows. UPFs and SSBs are a core part of the U.S. food system continuing to drive chronic disease prevalence and severity, while disproportionally impacting lower income households. The U.S. must begin to impactfully address the abundance of these extremely unhealthy food products while better supporting the food systems capable of producing minimally processed, environmentally sustainable and nutrient rich F/V sources for dietary health guideline compliance. U.S. food policy must be tailored to address key SDOH driving health disparities. The lack of nutritious and affordable foods in lower income areas are due to supply chain inadequacies manifesting as food deserts and food swamps overridden with cheap UPFs. U.S. food policy also must account for rapidly altered post-COVID-19 digital landscapes, technological healthcare and commercial agriculture advances, in order to foster food industry and benefits management innovations that ensure no one is left behind. Learning from COVID-19’s food supply chain lessons, the 2023–24 Farm Bill renewal presents a valuable time to implement robust and impactful U.S. SNAP benefit modernization to reduce the rates of diet-related chronic disease health disparities, support low-income communities, strengthen access to sustainable, resilient, climate friendly food systems, and implement “food is medicine” approaches.

Using a reputable national policy analysis framework tool, this policy brief provides cutting edge, evidence-based policy solutions, in direct consideration for the 2023–24 Farm Bill Reauthorization. Our findings and recommendations are aligned with the bipartisan 2022 White House Council on Hunger Health and Nutrition, the first conference on this topic in over 50 years. Policy recommendations are not just based in scientific evidence, but are also in direct alignment with the political feasibility and stated preferences of a number of bipartisan stakeholders. The crucial issues identified by this food policy analysis project support a number of key strategic priority areas for advancing U.S. human health and nutrition including: achieving racial and nutritional equity, reducing healthcare costs for diet-related chronic diseases, supporting post-COVID-19 recovery and supply chain innovation and growth, and promoting climate-smart agriculture for specialty crops.

## Author contributions

EM: Conceptualization, Formal analysis, Investigation, Methodology, Writing – original draft, Writing – review & editing. EK-T: Conceptualization, Course design, Formal analysis, Investigation, Methodology, Project administration, Resources, Supervision, Writing – original draft, Writing – review & editing.
